# Diaphragmatic ultrasonography-based rapid shallow breathing index for predicting weaning outcome during a pressure support ventilation spontaneous breathing trial

**DOI:** 10.1186/s12890-022-02133-5

**Published:** 2022-09-07

**Authors:** Jia Song, Zhixian Qian, Haixiang Zhang, Minjia Wang, Yihua Yu, Cong Ye, Weihang Hu, Shijin Gong

**Affiliations:** 1 Department of Critical Care Medicine, Zhejiang Hospital, 12 Lingyin Road, Xihu District, Hangzhou, 310013 Zhejiang China; 2 Department of Cardiovascular Medicine, Xinchang People’s Hospital, No. 117, Gushan Road, Nanming St, Xinchang, 312500 China; 3 Department of Gastroenterology and Hepatology, The First People’s Hospital of Xiaoshan District, No. 199, Shixin Road, Xiaoshan District, Hangzhou, 311203 China

**Keywords:** Diaphragmatic ultrasonography, Weaning from mechanical ventilation, Spontaneous breathing trial, Rapid shallow breathing, Diaphragmatic excursion, Diaphragm thickening fraction

## Abstract

**Background:**

The optimum timing to wean is crucial to avoid negative outcomes for mechanically ventilated patients. The rapid shallow breathing index (RSBI), a widely used weaning index, has limitations in predicting weaning outcomes. By replacing the tidal volume of the RSBI with diaphragmatic excursion (DE) and diaphragm thickening fraction (DTF) assessed by ultrasonography, we calculated two weaning indices, the diaphragmatic excursion rapid shallow breathing index (DE-RSBI, respiratory rate [RR]/DE) and the diaphragm thickening fraction rapid shallow breathing index (DTF-RSBI, RR/DTF). The aim of this study was to evaluate the predictive values of DTF-RSBI, DE-RSBI and traditional RSBI in weaning failure.

**Methods:**

This prospective observational study included patients undergoing mechanical ventilation (MV) for > 48 h and who were readied for weaning. During a pressure support ventilation (PSV) spontaneous breathing trial (SBT), right hemidiaphragmatic excursion and DTF were measured by bedside ultrasonography as well as RSBI. Weaning failure was defined as: (1) failing the SBT and (2) SBT success but inability to maintain spontaneous breathing for more than 48 h without noninvasive or invasive ventilation. A receiver operator characteristic (ROC) curve was used for analyzing the diagnostic accuracy of RSBI, DE-RSBI, and DTF-RSBI.

**Results:**

Of the 110 patients studied, 37 patients (33.6%) failed weaning. The area under the ROC (AUROC) curves for RSBI, DE-RSBI, and DTF-RSBI for predicting failed weaning were 0.639, 0.813, and 0.859, respectively. The AUROC curves for DE-RSBI and DTF-RSBI were significantly higher than for RSBI (*P* = 0.004 and *P* < 0.001, respectively). The best cut-off values for predicting failed weaning were RSBI > 51.2 breaths/min/L, DE-RSBI > 1.38 breaths/min/mm, and DTF-RSBI > 78.1 breaths/min/%.

**Conclusions:**

In this study, two weaning indices determined by bedside ultrasonography, the DE-RSBI (RR/DE) and DTF-RSBI (RR/DTF), were shown to be more accurate than the traditional RSBI (RR/VT) in predicting weaning outcome during a PSV SBT.

**Supplementary Information:**

The online version contains supplementary material available at 10.1186/s12890-022-02133-5.

## Background

Mechanical ventilation (MV) is used to sustain respiratory function in patients with acute respiratory failure. When the cause of acute respiratory failure improves, MV should be discontinued as soon as possible. Both early and delayed weaning are associated with increased mortality, intensive care unit (ICU) stay, and economic cost [1–3]. Therefore, determining the optimum time to wean mechanically ventilated patients is of paramount importance to improve these patients’ outcomes in the ICU. Nevertheless, deciding when to wean patients from MV can be challenging for intensivists [4].

Currently, many indices and parameters have been developed to assess a patient’s ability to breathe spontaneously. Rapid shallow breathing index (RSBI), which is calculated by dividing respiratory rate (RR) by tidal volume (VT), is the most commonly measured index [5] to predict weaning outcome. During a weaning attempt, RSBI measures the balance between the mechanical load on the inspiratory muscles and the inspiratory muscles’ ability to respond to this load [6]. Nonetheless, its low specificity and positive predictive value (PPV) can still lead to errors in weaning assessment [7, 8].

The diaphragm is the principal respiratory muscle and plays a crucial role in generating VT in healthy subjects [9]. From previous studies, diaphragmatic dysfunction (DD) is a common occurrence and has likely been underestimated in critically ill patients [10–12]. Thus, evaluating diaphragmatic function before any weaning attempt seems essential. Diaphragmatic ultrasonography has been recently proposed as a simple, non-invasive bedside method to assess the functional status of the diaphragm [13, 14]. There are two proposed diaphragmatic ultrasonography predictors: diaphragmatic excursion (DE) and diaphragm thickening fraction (DTF). Interestingly, in a prospective observational study [15], Spadaro et al. substituted VT with DE in the RSBI, calculating a new parameter, the diaphragmatic-RSBI (D-RSBI, RR/DE), and compared the ability of traditional RSBI and D-RSBI to predict weaning failure during a T-piece spontaneous breathing trial (SBT). The results demonstrated that D-RSBI was more accurate than traditional RSBI in predicting the weaning outcome. However, according to our experience, DE is affected by many factors, from the patient’s breathing status to the weaning mode, as well as intra-thoracic and intra-abdominal pressures. DTF is influenced by active contraction, and DTF performs better than DE when evaluating the diaphragmatic function [16].

In our study, we replaced VT in the RSBI with DE and DTF, respectively, when calculating two indices: diaphragmatic excursion rapid shallow breathing index (DE-RSBI, RR/DE) and diaphragm thickening fraction rapid shallow breathing index (DTF-RSBI, RR/DTF). We conducted this prospective study to evaluate the diagnostic performance of DE-RSBI, DTF-RSBI and conventional RSBI for predicting weaning outcome.

## Materials and methods

### Patient enrolment

This prospective observational study was performed from June 2017 to May 2018 in the ICU of a tertiary hospital in Zhejiang, China. The study was approved by the ethics committee of our institution (protocol number: 201610K), and the study was conducted according to the tenets of the Declaration of Helsinki. Written informed consent was obtained from each patient’s next of kin prior to participation. Patients were enrolled if they underwent invasive MV for more than 48 h and met all of the following criteria for an SBT: (1) resolution or improvement of the disease leading to MV; (2) adequate oxygenation, indicated by arterial oxygen saturation (SaO_2_) > 90% with inspired oxygen fraction (FiO_2_) ≤ 0.5, or arterial oxygen partial pressure to inspired oxygen fraction (PaO_2_/FiO_2_) ≥ 150 mmHg, both with positive end-expiratory pressure (PEEP) ≤ 8 cmH_2_O; (3) adequate pulmonary function, indicated by a RR < 30 breaths/min with VT ≥ 5 mL/kg ideal body weight (IBW) and no significant respiratory acidosis; (4) stable hemodynamics status, indicated by a systolic arterial blood pressure of 90–160 mmHg without or with minimal vasopressors (dopamine or dobutamine < 5 μg/kg/min or norepinephrine < 0.05 μg/kg/min) and heart rate (HR) < 120 beats/min; (5) adequate consciousness without sedation; (6) absence of excessive tracheobronchial secretion; and (7) effective cough reflex [17].

The exclusion criteria were as follows: (1) age < 18 years; (2) pregnancy; (3) presence of thoracostomy, pneumothorax, or pneumomediastinum; (4) presence of flail chest or rib fractures; (5) pre-existing cervical spinal injury, history or final diagnosis of neuromuscular disorders; (6) use of neuromuscular blocking agents within 48 h preceding the diaphragm function assessment; (7) history or new detection of paralysis (no movement) or paradoxical movement of a single hemidiaphragm on diaphragmatic ultrasonography; (8) the quality of the diaphragmatic ultrasonography images is poor and cannot be used for analysis; and (9) patient’s next of kin refused participation.

### Study design

Patients who met the inclusion criteria underwent a SBT using a pressure support ventilation (PSV) strategy, which uses a pressure support of 8 cmH_2_O and zero PEEP [18]. The duration was 30 min and the FiO_2_ was set at the same level (≤ 0.5) used during MV. After successfully passing the SBT, the physicians in charge (who were blinded to the diaphragmatic ultrasonography parameters) decided whether to extubate or wean from the ventilator (patients with tracheotomy). Weaning failure was defined as: (1) failing the SBT and (2) SBT success but inability to maintain spontaneous breathing for more than 48 h without noninvasive or invasive ventilation. Criteria for failure on the SBT were as follows: (1) acute respiratory distress (RR > 35 breaths/min); (2) SaO_2_ < 90% with an FiO_2_ ≥ 50%; (3) HR > 140 beats/min or an increase of ≥ 20%; (4) systolic arterial blood pressure ≥ 180 mmHg or an increase of ≥ 20%; and (5) change in mental status, agitation or anxiety [19].

As one of the ventilator parameters, RSBI was recorded as the value displayed by the ventilator (V500 and Savina 300; Drager Medical, Germany) with a flow-by technique, using the following ventilator settings: pressure support of 0 cmH_2_O, PEEP of 0 cmH_2_O, flow trigger of 2 L/min, and bias flow of 10 L/min [20]. Ultrasonographic images were acquired after 30 min from the beginning of the SBT, or immediately before returning to their initial ventilator settings in the case of SBT failure. The DE-RSBI and DTF-RSBI were calculated as the ratio of RR at the end of SBT to DE and DTF, respectively.

### Diaphragmatic ultrasonography measurements

**Fig. 1:**
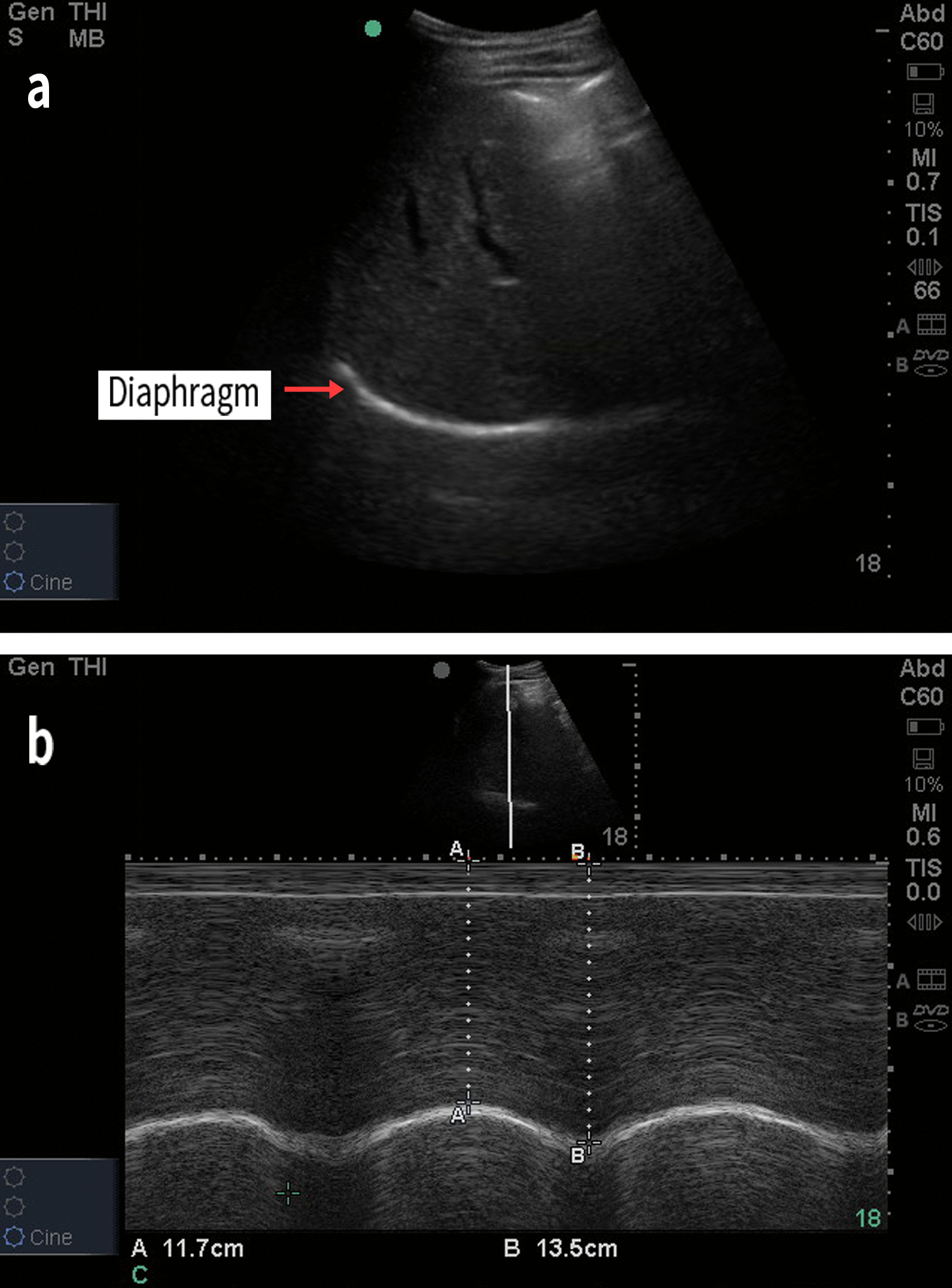
Diaphragmatic excursion (DE) measurement. **a** B-mode diaphragmatic ultrasonography. The bright line reflects the diaphragm. **b** M-mode diaphragmatic ultrasonography. DE during inspiration (A) and expiration (B) can be calculated according to B–A. In this image, DE was calculated as: 13.5 − 11.7 = 1.8 cm

**Fig. 2:**
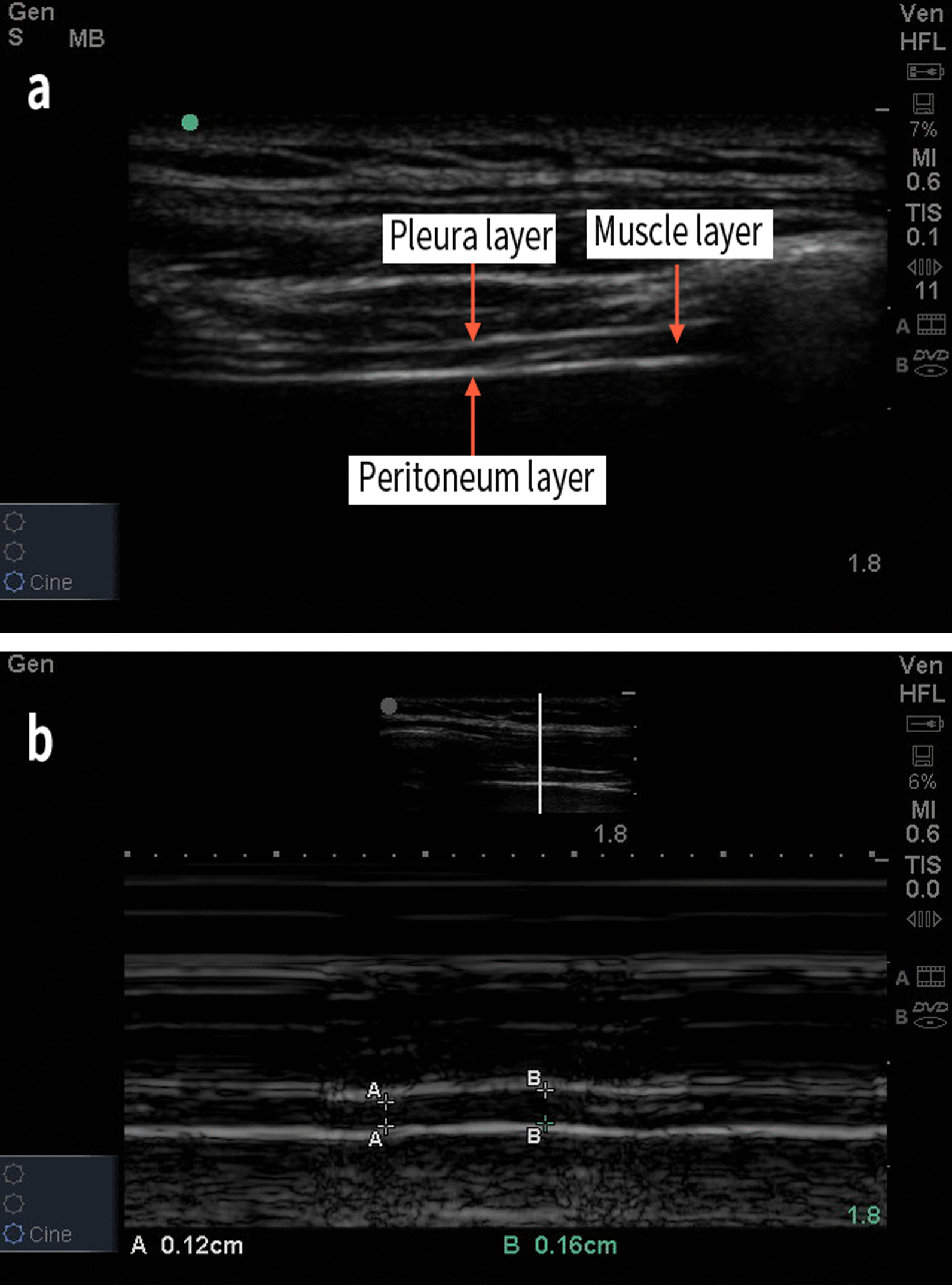
Diaphragm thickness (DT) measurement. **a** B-mode ultrasonography of the diaphragm in the zone of apposition. DT is a measurement of the muscle layer between the pleural layer and the peritoneal layer. **b** M-mode ultrasonography of the diaphragm in the zone of apposition. DT is measured at both end-inspiration (B) and end-expiration (A). In this image, diaphragm thickening fraction (DTF) was calculated as: 1.6 − 1.2/1.2 × 100% = 33.3%

The technique for ultrasonographic assessment of the DE (Fig. 1) and the diaphragm thickness (DT) (Fig. 2) are described in detail in Additional file.

### Assessment of the reproducibility of the ultrasonographic parameters

Thirty patients were randomly selected to assess reproducibility. In the inter-observer reproducibility study, two intensivists (JS and MJW) measured DE and DTF in the same sample of patients, with a time difference of less than 30 min between the two operators. The operators were blinded to each other’s findings. To assess intra-observer reproducibility, one of the operators (JS) repeated the measurement 5 min after the initial measurement.

### Statistical analysis

Continuous data are expressed as mean (± SD) and/or median (interquartile range) according to their distribution (Kolmogorov–Smirnov test). Categorical variables were expressed as numbers and percentages. Two means were compared with Student’s *t*-test or the Mann–Whitney U test, and two proportions were compared with the chi-square test or Fisher’s exact test, as appropriate. Receiver operating characteristic (ROC) curves were constructed to evaluate the performance of the five indices (RSBI, DE, DTF, DE-RSBI, and DTF-RSBI) to predict weaning failure. Sensitivities, specificities, positive predictive values (PPV), negative predictive values (NPV), positive and negative likelihood ratios were calculated. The best threshold value for each index was determined as the value associated with the best Youden’s index for the prediction of weaning failure. The comparison of the area under the ROC (AUROC) curves for RSBI, DE-RSBI, and DTF-RSBI was performed as described by DeLong et al. [21]. A two-tailed *P* value ≤ 0.05 was considered statistically significant.

The sample size was calculated considering an AUROC of more than 0.80 as acceptable diagnostic accuracy. According to the study by Spadaro et al. [15], assuming a prevalence of 33% weaning failure. Using a Type I error of 0.05 and a Type II error of 0.1 (power is 90%), a minimal sample size of 49 patients was calculated. After estimating a 10% dropout rate, a minimal sample size of 55 patients was required.

A multivariate logistic regression model was used to analyze the association between DE-RSBI, DTF-RSBI and weaning failure, after adjusting for confounders (age, sex, APACHE II score, length of MV until SBT and RR prior to SBT).

The reproducibility of DE and DTF measurements were expressed as the intra-class correlation coefficient (ICC). Statistical analyses were performed using SPSS 20.0 statistical software (IBM Corp., Armonk, NY, USA).

## Results

### Baseline characteristics of patients

**Fig. 3:**
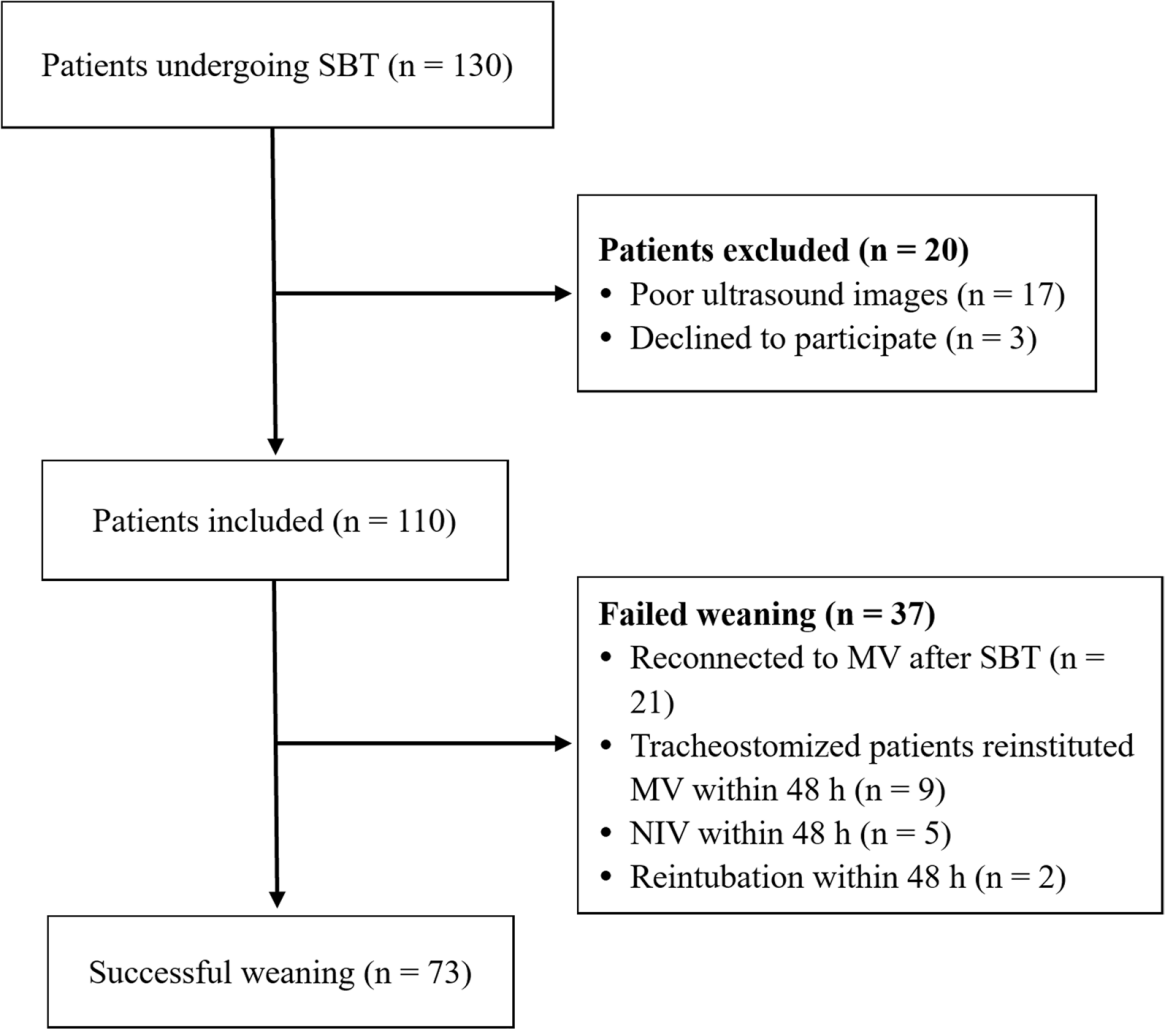
Study flow chart. *MV* mechanical ventilation, *SBT* spontaneous breathing trial, *NIV* non-invasive ventilation

During the study period, 130 patients were enrolled, but 20 were excluded because of poor ultrasonographic images (n = 17) and declined to participate (n = 3). Of the 110 patients included, 73 patients (66.4%) passed the SBT and were successfully weaned from MV. Among the patients who failed the weaning (n = 37), 21 (19.1%) failed the SBT, and 16 (14.5%) passed the SBT but breathed spontaneously for less than 48 h (2 were reintubated, 5 received non-invasive ventilation, and 9 tracheostomized patients were reinstituted to MV) (Fig. 3). Sixteen patients with tracheostomy were included (acute exacerbations of chronic obstructive pulmonary disease [n = 5], pneumonia [n = 5], acute stroke [n = 4] and traumatic brain injury [n = 2]). Of the tracheostomized patients, seven (43.8%) successfully weaned from MV, while 9 (56.2%) were reinstituted to MV within 48 h.

Demographic characteristics, blood gas analysis results, and ventilator parameters prior to SBT were not significantly different between the weaning success and failure groups (Table 1). The RR prior to SBT was significantly lower in patients who were successfully weaned than in those who failed the weaning attempt (*P* = 0.015). Patients who were successfully weaned had significantly lower lengths of ICU and hospital stay than those who failed weaning (*P* < 0.001, *P* = 0.001, respectively).

**Table 1:** Patient characteristics

Variables	All (n = 110)	Weaning success (n = 73)	Weaning failure (n = 37)	*P* value
Age, years	71.3 ± 17.3	70 ± 19	73.8 ± 13.4	0.291
Male, *n* (%)	78 (70.9)	52 (71.2)	26 (70.3)	0.916
APACHE II score	17 [15–20]	17 [15–20]	16 [15–18.5]	0.268
Length of MV until SBT, day	5.5 [3–9]	5 [3–8]	7 [4–9.5]	0.163
HR, beats/min	85.8 ± 13.7	85.1 ± 13.1	87.3 ± 15.1	0.429
RR prior to SBT, breaths/min	17.4 ± 3.4	16.9 ± 2.9	18.5 ± 3.9	0.015
MAP, mmHg	87 [77–95.5]	89 [77.5–95]	84 [74–99]	0.253
Reason for MV, *n* (%)				
Pneumonia	30 (27.3)	20 (27.4)	10 (27)	0.967
AECOPD	22 (20)	13 (17.8)	9 (24.3)	0.42
Heart failure	12 (10.9)	9 (12.3)	3 (8.1)	0.728
Septic shock	8 (7.3)	5 (6.8)	3 (8.1)	0.882
Traumatic brain injury	8 (7.3)	6 (8.2)	2 (5.4)	0.882
Poisoning	3 (2.7)	2 (2.7)	1 (2.7)	0.543
Postsurgery	5 (4.5)	4 (5.5)	1 (2.7)	0.86
Acute stroke	13 (11.8)	8 (11)	5 (13.5)	0.937
CPR	2 (1.8)	1 (1.4)	1 (2.7)	–
Hemorrhagic shock	5 (4.5)	4 (5.5)	1 (2.7)	0.86
Others	2 (1.8)	1 (1.4)	1 (2.7)	–
Blood gas analysis prior to SBT				
PaO_2_ (mmHg)	107.5 [98–130.3]	108 [99–124.5]	106 [95.5–147.5]	0.862
PaCO_2_ (mmHg)	37.5 [30, 31, 33–41]	37 [31–41.5]	39 [33–46]	0.204
PaO_2_/FiO_2_ (mmHg)	300 [254.4–350.4]	300 [253.8–340]	312.5 [250–383.3]	0.423
MV parameters prior to SBT				
PS, cmH_2_O	14 [12–15]	13 [12–15]	14 [11–15.5]	0.847
PEEP, cmH_2_O	3 [3, 4]	3 [3, 4]	4 [3, 4]	0.525
VT, ml	427.5 [408–508.3]	427 [408.5–514.5]	434 [398.5–503]	0.912
Clinical outcomes				
ICU length of stay, day	10 [6–15]	9 [5–13.5]	14 [10–17]	< 0.001
Hospital length of stay, day	17 [12–21, 32]	15 [10–20.5]	19 [16.5–23]	0.001

*APACHE II* acute physiology and chronic health evaluation II, *HR* heart rate, *RR* respiratory rate, *SBT* spontaneous breathing trial, *MAP* mean arterial pressure, *MV* mechanical ventilation, *AECOPD* acute exacerbations of chronic obstructive pulmonary disease, *CPR* cardiopulmonary resuscitation, *PaO*_*2*_*/FiO*_*2*_ arterial oxygen partial pressure to inspired oxygen fraction, *PS* pressure support, *PEEP* positive end-expiratory pressure, *VT* tidal volume

### Comparison of weaning parameters between patients with weaning success and failure

**Fig. 4:**
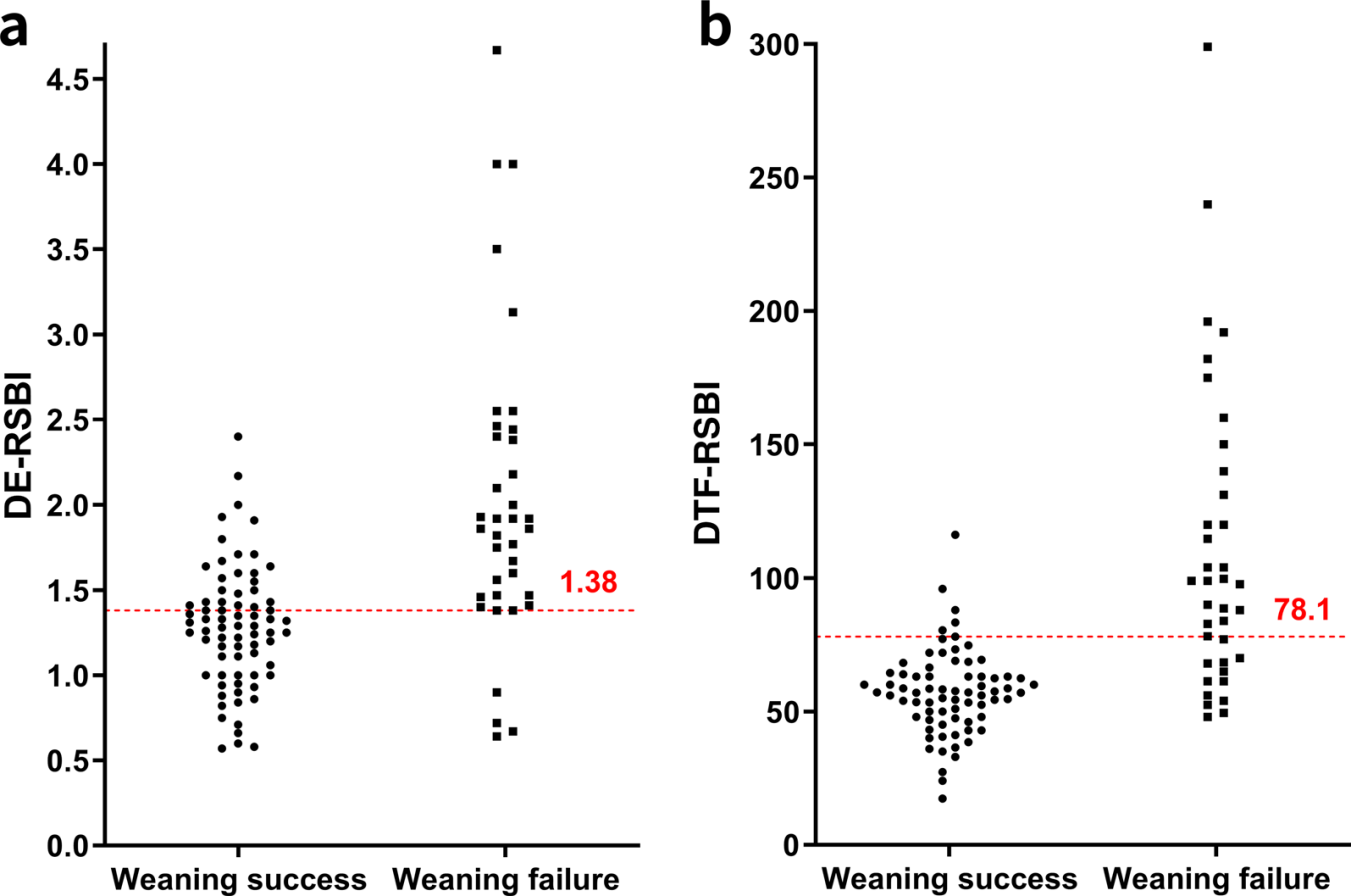
Dot plot of the diaphragmatic excursion rapid shallow breathing index (DE-RSBI) (**a**) and the diaphragm thickening fraction rapid shallow breathing index (DTF-RSBI) (**b**) in weaning success and failure groups

Significant differences in DE, DTF, DE-RSBI, and DTF-RSBI were observed between the weaning success and failure groups (*P* < 0.001; Fig. 4; Table 2). In addition, other parameters displayed statistically significant differences between the success and failure groups, namely RR at the end of the SBT and RSBI (*P* = 0.005, *P* = 0.018, respectively).

**Table 2:** Comparison of weaning parameters between patients with weaning success vs failure

Variables	All (n = 110)	Weaning success (n = 73)	Weaning failure (n = 37)	*P* value
RR at the end of SBT, breaths/min	22 ± 4.3	21.2 ± 3.7	23.5 ± 4.9	0.005
VT, ml	446 ± 107.1	456.1 ± 109.7	426 ± 101.9	0.167
PaO_2_/FiO_2_ (mmHg)	271.1 [216.7–332.7]	277.5 [222.5–321.3]	248.6 [206.6–345.2]	0.371
RSBI, breaths/min/L	48.5 [37.7–59.8]	46.2 [37.5–55.6]	53.6 [39.9–70.6]	0.018
DE, mm	16 [13–19]	17 [14.5–19.5]	12 [10–15.5]	< 0.001
DT at end inspiration, mm	2.1 [1.8–2.4]	2.1 [1.8–2.4]	2.1 [1.8–2.4]	0.493
DT at end expiration, mm	1.5 [1.4–1.8]	1.5 [1.3–1.8]	1.6 [1.4–2]	0.27
DTF, %	33.3 [26.3–40]	37.5 [33.3–41.9]	23.1 [19.5–29.4]	< 0.001
DE-RSBI, breaths/min/mm	1.4 [1.2–1.8]	1.3 [1–1.5]	1.9 [1.5–2.4]	< 0.001
DTF-RSBI, breaths/min/%	61.9 [53.1–82.9]	57.2 [47.8–63.5]	97.8 [68.2–135.6]	< 0.001

*RR* respiratory rate, *SBT* spontaneous breathing trial, *VT* tidal volume, *PaO*_*2*_*/FiO*_*2*_ arterial oxygen partial pressure to inspired oxygen fraction, *RSBI* rapid shallow breathing index, *DE* diaphragmatic excursion, *DT* diaphragm thickness, *DTF* diaphragm thickening fraction, *DE-RSBI* diaphragmatic excursion rapid shallow breathing index, *DTF-RSBI* diaphragm thickening fraction rapid shallow breathing index

### Predictive value of RSBI, DE, DTF, DE-RSBI and DTF-RSBI for weaning failure

**Fig. 5:**
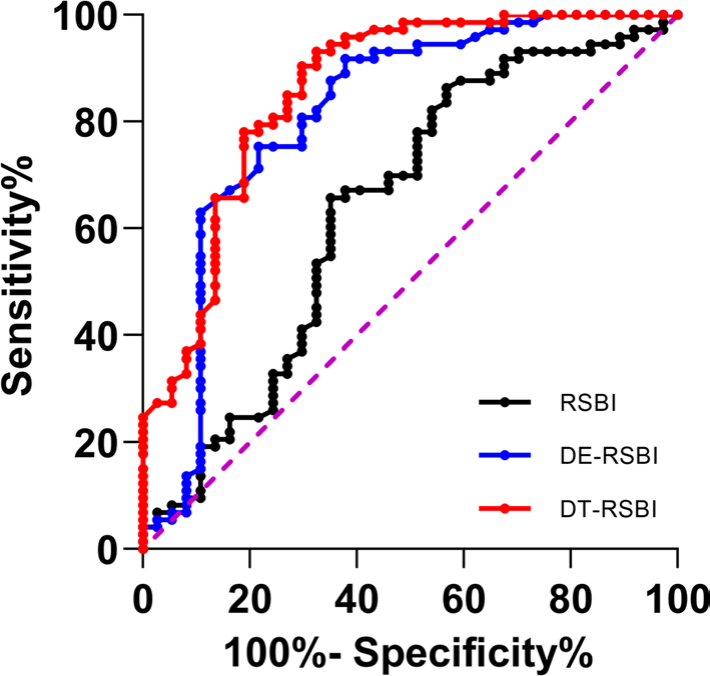
Receiver operating characteristic curves to predict weaning failure using the rapid shallow breathing index (RSBI), diaphragmatic excursion rapid shallow breathing index (DE-RSBI), and diaphragm thickening fraction rapid shallow breathing index (DTF-RSBI)

A ROC curve was used to assess the diagnostic accuracy of the weaning parameters in predicting weaning failure from MV (Fig. 5). The AUROCs for RSBI, DE, DTF, DE-RSBI, and DTF-RSBI were 0.639 (95% confidence interval [CI] 0.522–0.756, *P* = 0.018), 0.771 (0.664–0.877, *P* < 0.001), 0.868 (0.792–0.944, *P* < 0.001), 0.813 (95% CI 0.716–0.91, *P* < 0.001), and 0.859 (95% CI 0.78–0.939, *P* < 0.001), respectively. The best cut-off values for predicting weaning failure were RSBI > 51.2 breaths/min/L, DE < 13.5 mm, DTF < 30.09%, DE-RSBI > 1.38 breaths/min/mm, and DTF-RSBI > 78.1 breaths/min/% (Table 3). It is worth noting that the AUROCs for DE-RSBI and DTF-RSBI were higher than for RSBI (*P* = 0.004, *P* < 0.001, respectively), but there was no statistical difference between DE-RSBI and DTF-RSBI (*P* = 0.348).

**Table 3:** Predictive value of RSBI, DE, DTF, DE-RSBI, and DTF-RSBI for weaning failure

Variables	Threshold	AUC (95% CI)	*P* value	Sensitivity (%)	Specificity (%)	PPV (%)	NPV (%)	LR +	LR −
RSBI, breaths/min/L	> 51.2	0.639 (0.522–0.756)	0.018	64.9	65.8	49	78.7	1.9	0.53
DE, mm	< 13.5	0.771 (0.664–0.877)	< 0.001	64.9	89	74.9	83.3	5.9	0.39
DTF, %	< 30.09	0.868 (0.792–0.944)	< 0.001	78.4	84.9	72.5	88.6	5.19	0.s25
DE-RSBI, breaths/min/mm	> 1.38	0.813 (0.716–0.91)	< 0.001	89.2	65.8	56.9	92.3	2.61	0.16
DTF-RSBI, breaths/min/%	> 78.1	0.859 (0.78–0.939)	< 0.001	67.6	93.2	83.4	85	9.94	0.35

*AUC* area under the curve, *CI* confidence interval, *PPV* positive predictive value, *NPV* negative predictive value, *LR* likelihood ratio, *RSBI* rapid shallow breathing index, *DE* diaphragmatic excursion, *DTF* diaphragm thickening fraction, *DE-RSBI* diaphragmatic excursion rapid shallow breathing index, *DTF-RSBI* diaphragm thickening fraction rapid shallow breathing index

### Independent predictors for weaning failure

**Fig. 6:**
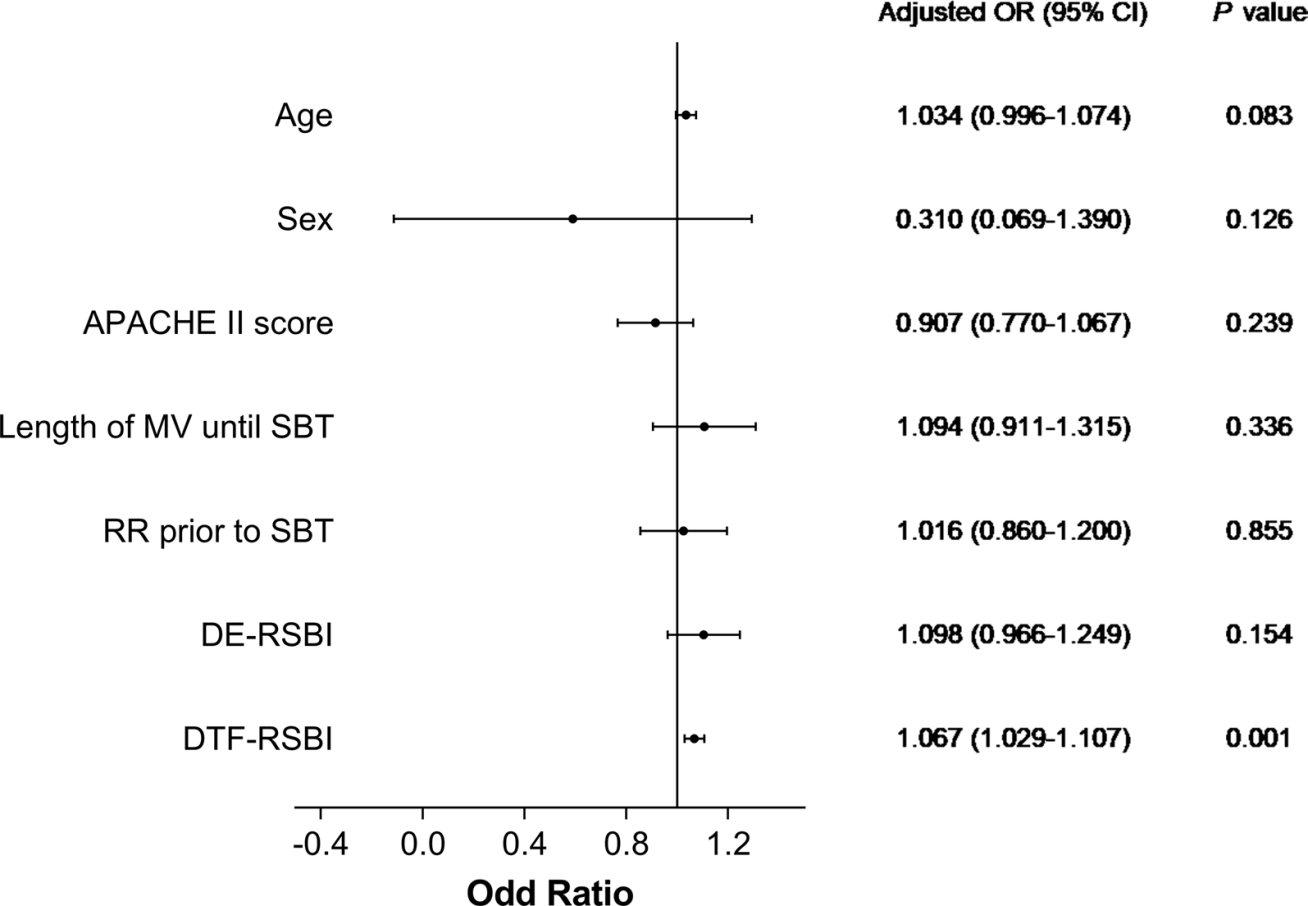
Forest plot of risk factors for weaning failure in multivariate logistic regression analysis. *OR* odds ratio, *CI* confidence interval, *APACHE II* acute physiology and chronic health evaluation II, *MV* mechanical ventilation, *SBT* spontaneous breathing trial, *RR* respiratory rate, *DE-RSBI* diaphragmatic excursion rapid shallow breathing index, *DTF-RSBI* diaphragm thickening fraction rapid shallow breathing index

In a multivariate logistic regression analysis, after adjusting for age, sex, APACHE II score, length of MV until SBT and RR prior to SBT, the DTF-RSBI was independently associated with weaning failure (odds ratio [OR] 1.067, 95% CI 1.029–1.107, *P* = 0.001) (Fig. 6).

### Reproducibility of DE and DTF

Intra-observer reliability ICCs for DE and DTF were 0.982 (95% CI 0.964–0.992, *P* < 0.001) and 0.905 (95% CI 0.812–0.953, *P* < 0.001), respectively. Inter-observer reliability ICCs for DE and DTF were 0.885 (95% CI 0.772–0.943, *P* < 0.001) and 0.815 (95% CI 0.647–0.907, *P* < 0.001), respectively.

## Discussion

The main finding of this study is that two weaning indices determined by bedside ultrasonography and RSBI, DE-RSBI (RR/DE) and DTF-RSBI (RR/DTF), are more accurate than the traditional RSBI (RR/VT) in predicting the weaning outcome during a PSV SBT.

The pathophysiology of weaning failure is complex, and it includes dysfunctional respiratory centers, respiratory muscle dysfunction, respiratory muscles overload, weaning-induced cardiovascular dysfunction, or a reduced ability to clear secretions [15–17]. An ideal predictive index should consider as many as pathophysiological pathways that may contribute to weaning failure. Various weaning indices have been investigated to identify an optimal weaning window, but no index has proven to be ideal. Among these indices, the RSBI, described as the ratio of RR to VT, has gained wide use owing to its simple technique. RSBI is a global index of weaning-induced patient distress, most commonly resulting from physiopathological mechanisms leading to breathing rapidly (high RR) and shallowly (low VT) [22]. Yang and Tobin, in their original prospective cohort study, found that an RSBI of < 105 breaths/min/L as a threshold for predicting extubation success with a sensitivity, specificity, PPV, and NPV of 97%, 64%, 78%, and 95%, respectively [6]. However, lower predictive values have been reported in other studies [8, 23]. The AUROC value for RSBI in our study was lower (0.639) compared with Yang and Tobin’s result (0.89). We speculate that this difference might be related to the RSBI measurement technique. In Yang and Tobin’s study, RSBI was measured by Wright spirometer and disconnected the patients from the ventilator. While, the value displayed by the ventilator during unassisted breathing (pressure support of 0 cmH_2_O and PEEP of 0 cmH_2_O) in our study. A recent study compared two RSBI measurement techniques (measured by the ventilator and Wright spirometer) in patients with readiness for weaning, the authors found that the ventilator significantly overestimates the RSBI value compared to the standard technique by Wright spirometer [20].

With the widespread use of ultrasonographic techniques in the ICU, diaphragmatic ultrasonography has received increasing attention. Ultrasonography allows both morphological assessment (detection of atrophy) and functional evaluation of the diaphragm (contractility). The measures of diaphragmatic function comprise DE and DTF, and DE is mainly related to the inspiratory volume during the inspiratory phase, regardless of whether it depends on muscle workload or ventilator support [24, 25]. DTF, also known as the “ejection fraction” of the diaphragm, reflects the active contraction ability of the diaphragm in the face of mechanical load [22, 26, 27]. In the present study, both DE and DTF exhibited a higher predictive value than the RSBI, which is in line with previous studies [22, 28].

When a patient undergoes an SBT, the diaphragm generates sufficient VT through increased work. In the presence of DD, the accessory inspiratory muscles can increase work to maintain VT for a limited period [29, 30]. In these circumstances, RSBI may be within the normal range because it measures VT generated by the respiratory muscles as a whole without compensating for the diaphragm's contribution [31]. However, the accessory inspiratory muscles are much less efficient and easily fatigued, their exhaustion may result in delayed weaning failure in following hours. The contribution of accessory muscles to VT may give rise to a false negative RSBI by masking the underlying DD. Hence, substituting DE and DTF for VT in the calculation of RSBI may be more accurate because DE and DTF has shown to reflect diaphragmatic function. This speculation was first confirmed in the research by Spadaro et al. [15]. In a prospective observational study, the authors simultaneously evaluated D-RSBI as well as the RSBI during a T-piece SBT, and reported that the AUROC values for D-RSBI and RSBI in predicting the weaning outcome were 0.89 and 0.72, respectively. In our study, both DE-RSBI and DTF-RSBI were significantly higher in patients who failed weaning compared with patients who were successfully weaned, and these indices showed a better performance than RSBI as a weaning predictor, with AUROC values of 0.814 and 0.859, respectively. These results demonstrate that DE-RSBI and DTF-RSBI are more accurate than RSBI in predicting the weaning outcome.

Importantly, we must clarify the following points before using this new index. First, under assisted modes of MV (e.g., PSV), DE is derived from adding patients’ respiratory effort to the pressure generated by the ventilator. In this case, distinguishing the effect of active diaphragmatic contraction on VT is complicated [32]. This explains why the DE-RSBI in the study by Spadaro et al. exhibited a higher predictive value than in our study. In their study, the mode of SBT was a T-piece, while the mode was PSV in our study. Second, DE may vary depending on a patient’s position, breathing pattern, and changes in abdominal and/or thoracic pressures (e.g., ascites, atelectasis) [33]. DE may exhibit higher values when patients are supine versus seated. Furthermore, deep, superficial, or irregular breaths cause measurement errors [34]. In contrast, DTF reflects variation in the thickness of the diaphragm during respiratory effort and it is influenced only by active contraction, regardless of whether the patient receives MV [35]. Recently, Llamas-Álvarez et al. suggested a lower accuracy for DE compared with DTF in predicting weaning outcome, and higher heterogeneity [16]. In our study, DTF-RSBI exhibited a higher predictive value than DE-RSBI. Considering the intrinsic deficiencies of DE, we believe that DTF-RSBI may be a better choice for predicting the weaning outcome when patients have undergone an SBT with PSV.

In the recent study [36], Fossat et al. proposed a new composite index named the rapid shallow diaphragmatic index (RSDI) (RSBI/DE). During a 30-min SBT with minimal PSV (pressure support of 6 cm H_2_O and end-expiratory pressure of 0 cm H_2_O), the diaphragmatic ultrasonography was performed and the RSDI, RSBI was calculated at the 5th and the 25th minute of the SBT. They found that the RSBI, other indices that incorporate the ultrasound mobility of the diaphragm into the calculation of the RSBI, the DE, and the DTF failed to predict the extubation or weaning success. The findings of the study are inconsistent with those of previous studies [15, 37], including the present study. Possible reasons for these differences may be explained as follows. First, Fossat et al. only analysed the patients who successfully passed SBT. Patients with SBT failure possibly due to DD were not included in the analysis. This may underestimate the value of diaphragm ultrasonography in predicting weaning outcomes. Second, in the study by Fossat et al. 25% of patients received prophylactic noninvasive ventilation after extubation. These patients were considered as weaning failures in our study. Prophylactic noninvasive ventilation may mask the postextubation respiratory failure caused by DD.

As ultrasonography is an operator-dependent method, minimizing intra-observer and inter-observer variation is very important. In our study, the ICC was evaluated to assess the intra-observer and inter-observer reproducibility of DE and DTF measurements. Consistent with previous studies [34, 38–40], we also found excellent reproducibility for DE and DTF measurements. In the present study, certain measures were used to decrease intra-observer and inter-observer variation. First, ultrasonography was performed by a well-trained point-of-care ultrasonography intensivist (JS) who had received more than 40 h of hands-on training in diaphragm ultrasonography and who had operating experience on more than 100 patients. Second, the patients’ posture was standardized. Third, the cursor for DE measurements in M-mode was kept as strictly perpendicular as possible regarding the middle or posterior part of the diaphragm, and for DT, a higher-resolution linear probe was used when necessary. Finally, every measurement was performed three times and then averaged.

The present study has several limitations. First, all measurements were made on the right hemidiaphragm as gastric or colic gas often impairs diaphragmatic imaging on the left side. Other investigators recommend that left hemidiaphragmatic measurements are unnecessary unless there is unilateral phrenic nerve injury [34, 41]. Second, neither echocardiography nor lung ultrasonography were evaluated in the patients with weaning failure. Such information could be helpful in determining whether weaning failure has its roots in DD or has a cardiac or a respiratory origin [42]. Therefore, comprehensive ultrasonographic assessment is required for patients with weaning failure. Third, additional limitations of this study are the small sample size and single-center enrollment. For these reasons, a prospective, randomized controlled, multicenter, large sample clinical study is required to establish the true predictive power of DE-RSBI and DTF-RSBI. Fourth, sixteen patients with tracheostomy were included in the present study. Tracheostomy patients received longer MV than intubated patients and it is known that prolonged MV can lead to diaphragmatic atrophy and contractile dysfunction. There are only few parameters that predict weaning outcomes in tracheostomy patients. Therefore, more prospective studies are required to focus on weaning predictors in this particular patient population. Finally, we must keep in mind that ultrasonography also has intrinsic limitations, especially a poor viewing window in obese patients, which may limit its use. Seventeen (13%) patients were excluded in our study due to a poor acoustic window.

## Conclusion

According to our results, during a PSV SBT, with DE and DTF measured by ultrasonography and replacing VT in the RSBI to calculate the two indices, DE-RSBI (RR/DE) and DTF-RSBI (RR/DTF), shown to me more accurate than the traditional RSBI (RR/VT) in predicting the weaning outcome. Point-of-care ultrasonography to assess diaphragmatic function had excellent reproducibility. To the best of our knowledge, the DTF-RSBI is first proposed and used to predict the weaning outcome, however, large prospective studies are required to validate the diagnostic accuracy of the index.

## Supplementary Information

**Additional file 1.** Description of the diaphragmatic ultrasonography measurements.

## Data Availability

The datasets used and/or analyzed during the current study are available from the corresponding author on reasonable request.

## Abbreviations

RSBI: Rapid shallow breathing index
DE: Diaphragmatic excursion
DTF: Diaphragm thickening fraction
MV: Mechanical ventilation
PSV: Pressure support ventilation
SBT: Spontaneous breathing trial
ROC: Receiver operating characteristic
ICU: Intensive care unit
PPV: Positive predictive value
VT: Tidal volume
DD: Diaphragmatic dysfunction
SaO_2_: Arterial oxygen saturation
FiO_2_: Inspiratory oxygen fraction
PaO_2_/FiO_2_: Arterial oxygen partial pressure to inspiratory oxygen fraction
PEEP: Positive end-expiratory pressure
RR: Respiratory rate
IBW: Ideal body weight
HR: Heart rate
DT: Diaphragmatic thickness
AUROC: Area under the receiver operating characteristic
ICC: Intra-class correlation coefficient
CI: Confidence interval
OR: Odds ratio
APACHE II: Acute physiology and chronic health evaluation II
MAP: Mean arterial pressure
AECOPD: Acute exacerbations of chronic obstructive pulmonary disease
CPR: Cardiopulmonary resuscitation
PS: Pressure support
NPV: Negative predictive value
LR: Likelihood ratio
