# Supplementary material for: Diaphragmatic ultrasonography-based rapid shallow breathing index for predicting weaning outcome during a pressure support ventilation spontaneous breathing trial
Source: BMC Pulm Med. 2022 Sep 7;22:337. doi: 10.1186/s12890-022-02133-5 (PMC9450260; doi:10.1186/s12890-022-02133-5)
Supplement: Supplementary file 1 — Additional file 1. Description of the diaphragmatic ultrasonography measurements. [file 12890_2022_2133_MOESM1_ESM.docx]

**Additional file**

**Diaphragmatic ultrasonography measurements**

The patients were in a semi-recumbent position, with the head of the bed elevated at an angle between 30° and 45°. Each measurement was repeated three times by a single well-trained intensivist (JS), and we used the mean value for analysis.

DE was measured with a 3.5-MHz convex phased array probe (M-turbo; FUJIFILM Sonosite, Washington, USA). Using the liver as an acoustic window, the probe was placed immediately below the right costal margin on the anterior axillary or midclavicular line in the longitudinal scanning plane, with the tomographic plane angled in the cephalad direction such that the ultrasound beam was perpendicular to the posterior third of the right hemidiaphragm. B-mode was used initially to obtain the best approach and select the exploration line; M-mode was then used to display the motion of the anatomical structures along the selected line. Normal inspiratory diaphragmatic movement is caudal because the diaphragm moves toward the probe; normal expiratory trace is cranial, as the diaphragm moves away from the probe(1). DE was measured on the vertical distance from the end of inspiration to the end of expiration (Fig. 1). As previously reported(2), the diaphragm was visualized by placing the 10-MHz linear probe perpendicular to the chest wall, in the eighth or ninth intercostal space, between the anterior axillary and midaxillary lines, to observe the zone of apposition of the muscle 0.5-2 cm below the costophrenic sinus. In this area, the diaphragm is observed as a structure comprised of three distinct layers in B-mode: a non-echogenic central layer (the muscle) bordered by two echogenic layers (the pleural line and the peritoneal line). To obtain adequate diaphragmatic images in M-mode, the diaphragm thickness (DT) was the muscle layer measurement at both end-inspiration and end-expiration during quiet spontaneous breathing (Fig. 2). Then, the DTF percentage was calculated from the following formula: (Thickness at end-inspiration − Thickness at end-expiration)/Thickness at end-expiration × 100%.

**References**

1. Matamis D, Soilemezi E, Tsagourias M, Akoumianaki E, Dimassi S, Boroli F, et al. Sonographic evaluation of the diaphragm in critically ill patients. Technique and clinical applications. Intensive Care Med. 2013;39(5):801-10.

2. Tuinman PR, Jonkman AH, Dres M, Shi ZH, Goligher EC, Goffi A, et al. Respiratory muscle ultrasonography: methodology, basic and advanced principles and clinical applications in ICU and ED patients-a narrative review. Intensive Care Med. 2020;46(4):594-605.
